# Optimization of butter, xylitol, and high‐amylose maize flour on developing a low‐sugar cookie

**DOI:** 10.1002/fsn3.1160

**Published:** 2019-09-18

**Authors:** Yunxian Song, Xu Li, Yuyue Zhong

**Affiliations:** ^1^ College of Life Science Huaibei Normal University Huaibei China; ^2^ College of Agronomy Northwest A&F University Yangling China

**Keywords:** butter, high‐amylose maize flour, low‐sugar cookie, response surface methodology, xylitol

## Abstract

There is a huge interest to develop low‐sugar baked products for reducing risks of some diseases, such as adiposis, diabetes, and high blood pressure. A low‐sugar cookie was prepared with butter, xylitol, and high‐amylose maize flour (HAMF) through response surface methodology. ANOVA of models for sensory profiles, texture, and digestibility showed the models for sensory attributes, hardness, and resistant starch were significant (*p* < .05), indicating the reliability of these models. Sensory profiles of cookie were mainly affected by butter and xylitol, while HAMF was not significant. Hardness was negatively related to butter and HAMF. Resistant starch (RS) content was positively correlated with butter, xylitol, and HAMF. The improvement of RS was attributed to high proportions of long amylopectin and amylose chains of starch in HAMF and interactions of starch with butter and xylitol. The predicted model showed the optimal combination of a cookie with the highest sensory and resistant starch and the lowest hardness was intermediate butter, high xylitol, and high HAMF contents.

## 1 INTRODUCTION

The consumption of high quantities of sugar contributes to some diseases, such as diabetes and high blood pressure. Therefore, there is a huge interest to improve food safety by developing low‐sugar foods. However, sugar is not easy to be decreased, due to its importance in some food systems, such as cookie. The major sugar sources in cookies are starch and sucrose (Pareyt & Delcour, 2008). Starch is the main component of flour, which is responsible for the texture and nutritional properties of cookie (Kaldy, Rubenthaler, Kereliuk, Berhow, & Vandercook, 1991). The sucrose is another important ingredient by providing texture and sweetness to the cookie (Zoulias, Oreopoulou, & Kounalaki, 2002).

The main strategy for developing low‐sugar bakery products is to find low‐sugar substitutions of normal wheat flour and sucrose (Giuberti, Gallo, Fortunati, & Rossi, 2016; Kutyła‐Kupidura et al., 2016; Luhovyy et al., 2014; Nourmohammadi & Peighambardoust, 2016). High‐amylose maize flour (HAMF) is one ideal substitute of normal wheat flour. High‐amylose starch can lower blood glucose level of humans by reducing digestible starch content (Luhovyy et al., 2014; Zhong et al., 2018). In addition, high‐amylose starch has a higher content of resistant starch (RS), which decreases plasma cholesterol and triglyceride concentrations and promotes the growth of beneficial microflora (Birt et al., 2013). HAMF‐based baked products, including bread, cakes, and cookies, can increase RS content and decrease glycemia levels in human body (Giuberti et al., 2016; Hung, Yamamori, & Morita, 2005; Zhong et al., 2018). On the other hand, due to the importance of gluten in wheat flour, an essential structure‐building protein, in providing viscoelasticity to the dough, good gas‐holding ability, and good crumb structure of bakery products (Gallagher, Gormley, & Arendt, 2004), HAMF is suggested as partial replacement of wheat flour, for maintaining structure and quality of products.

However, a potential risk of using high‐amylose starch on bakery products is the increase in hardness and the decrease in sensory properties of these products (Giuberti et al., 2016; Hung et al., 2005; Zhong et al., 2018). It is expected the detrimental effects of high‐amylose starch on sensory attributes can be mitigated by other ingredients. A strategy is to supplement butter in recipe of high‐amylose starch cookie, due to the function of butter in improving sensory attributes by affecting interactions between protein and other ingredients (Zoulias, Oreopoulou, & Kounalaki, 2002) and decreasing hardness of bakery foods (Pareyt & Delcour, 2008). To further lower blood glucose level, xylitol was added as the sucrose replace. Xylitol, a noncariogenic sweetener with the sweetness of 40%–100% that of sucrose (Grabitske & Slavin, 2008), can provide similar sensory attributes and texture as sucrose (Kutyła‐Kupidura et al., 2016; Mushtaq, Rehman, Zahoor, & Jamil, 2010; Ronda, Gómez, Blanco, & Caballero, 2005; Winkelhausen, Jovanovic‐Malinovska, Velickova, & Kuzmanova, 2007) and increase the density, porosity, and volume of bakery foods (Nourmohammadi & Peighambardoust, 2016).

Therefore, a novel low‐sugar cookie with acceptable sensory and texture attributes is expected to be developed by combining butter, xylitol, and HAMF. The individual effects of these three ingredients in bakery foods have been already studied (Giuberti et al., 2016; Pareyt & Delcour, 2008; Ur‐Rehman, Mushtaq, Zahoor, Jamil, & Murtaza, 2015); however, the effects of these factors in a complex bakery food system are not independent because of the strong interactions and competitions of different ingredients in this system (Fustier, Castaigne, Turgeon, & Biliaderis, 2008; Pareyt & Delcour, 2008). The purpose of this study was to explore the effects of butter, xylitol, and HAMF on sensory attributes, texture, and digestibility of cookie. For this purpose, response surface methodology (RSM), an effective way to study the relationships between one or more responses (dependent variables) and factors (independent variables) and to optimize the ingredient levels from raw to final products (Battaiotto, Lupano, & Bevilacqua, 2013), is adopted here. Butter, xylitol, and HAMF were set as three factors, and sensory attributes, texture, and digestibility were set as responses. It was expected that a novel low‐sugar cookie with acceptable sensory attributes is developed by butter, xylitol, and HAMF.

## 2 MATERIALS AND METHODS

### 2.1 Materials

Flour and starch from normal wheat and high‐amylose maize grains were obtained from Key Laboratory of Biology and Genetic Improvement of Maize in Arid Area of Northwest Region, Ministry of Agriculture, College of Agronomy, Northwest A&F University, Yangling, Shaanxi, China. Butter, xylitol, and eggs were purchased from Luwang Company. High‐amylose maize flour and starch were defined as HAMF and HAMS, separately. Pancreatin from porcine pancreas (Cat. No. P7545) and amyloglucosidase (Cat. No. A7095, activity 300 unit/ml) were purchased from Sigma‐Aldrich Chemical Co. Glucose oxidase–peroxidase (GOPOD) was from Megazyme Company, Ireland.

### 2.2 Molecular structure of high‐amylose starch

The weight size distributions of debranched normal wheat starch (NWS) and high‐amylose maize starch (HAMS) were analyzed by gel permeation chromatography (GPC) system (Agilent 1260 series, Agilent Technologies) equipped with a refractive index detector (Optilab T‐rEX; WYATT Corp.) and a differential pressure detector (Viscostar II; WYATT Corp.), by the method of (Li, Prakash, Nicholson, Fitzgerald, & Gilbert, 2016). Debranching of starch was conducted by the isoamylase as described (Kuang, Xu, Wang, Zhou, & Liu, 2016). Weight distribution, W(logV_h_), was plotted to present the molecular size distribution of samples, and the degree of polymerization (DP X) of debranched starch was calculated following the method described (Li et al., 2016).

### 2.3 Preparation of cookie

High‐amylose maize flour (HAMF) and normal wheat flour (NWF) passing through 180‐μm sieve were used. The dough was prepared with 500 g of mixed flour, 50–150 g xylitol, two eggs, and distilled water, to get final hydration of 24% (dough basis). According to the experimental design, the amount of HAMF addition was ranged from 150 g to 350 g. After mixing butter with liquid ingredients (two eggs, xylitol, and water), a liquid mixture was creamed and mixed with flour. The dough was then stirred in a domestic blender for 5 min before standing the dough for 20 min. After laminating the dough to a height of 2 mm using a pasta roller attachment, the dough was cut into circles with a circular mold (3 cm diameter) and baked in a baking oven at 180°C for 18 min. Finally, the cookies were cooled for 2 hr and stored in different airtight plastic bags at room temperature.

### 2.4 Sensory evaluation of cookie

A 40‐member untrained panel (20 males and 20 females between 20 and 40 years old) was used to evaluate sensory profiles: the appearance, color, flavor, taste, mouthfeel, and overall acceptability of cookies using a nine‐point hedonic scale by filling out a scoring form with scores from 1 to 9.

#### 2.4.1 Appearance

A score of one point indicates the surface of the cookie is extremely rough/the cookie is incomplete; a score of nine points indicates the surface of the cookie is extremely smooth/the cookie is complete.

#### 2.4.2 Color

A score of one point indicates the distribution of color in the cookie is not even/the cookie is dark brown; a score of nine points indicates the distribution of color in the cookie is even/the cookie is golden yellow.

#### 2.4.3 Flavor

A score of one point indicates the flavor of the cookie is extremely faint; a score of nine points indicates the flavor of the cookie is extremely strong.

#### 2.4.4 Taste

A score of one point indicates the cookie is too greasy and the sweetness is not appropriate; a score of nine points indicates the cookie is not greasy and the sweetness is appropriate.

#### 2.4.5 Mouthfeel

A score of one point indicates the cookie is too hard and the mouthfeel is rough; a score of nine points indicates the cookie is crispy and the mouthfeel is delicate.

#### 2.4.6 Overall acceptability

A score of one point indicates extremely dislike; a score of nine points indicates extremely like.

### 2.5 Texture of cookie

The texture of cookies was determined using a texture analyzer (TVT 6700; Perten Swiss) and a single‐cycle compression test. A P‐BP70A probe and a R‐TPBR pedestal were used. The parameters were set as follows: 80% compression degree, 2.5 mm/s pretest speed, 2.0 mm/s test speed, 10.0 mm/s retraction speed, and 20 g trigger force. The hardness and flexibility of the cookies were determined using TexCalc software in the texture analyzer. Five different cookies were conducted for each trial in the RSM.

### 2.6 Digestibility of cookie

The cookies were ground and filtered through a 1.0‐mm sieve, and the contents of soluble starch (SS) and resistant starch (RS) were measured using AACC method (McCleary, Sloane, & Draga, 2015). The first step was to hydrolyze cookie by pancreatic α‐amylase and amyloglucosidase (AMG) at 37°C. Then, SS and RS were determined separately. RS was dissolved in KOH solution and then mixed with sodium acetate buffer before adding AMG to hydrolyze the RS. The determination of SS was performed by prewashing cookies by 8 ml of aqueous industrial methylated spirits (IMS) (80% v/v) before adding pancreatic α‐amylase and AMG. Then, GOPOD assay was used to measure the hydrolyzed glucose content of RS and SS, respectively, and transferred to RS and SS content. Triplicate was conducted for each formula.

### 2.7 Experimental design and statistical analysis

Response surface methodology was adopted to evaluate the effects of three independent variables, butter (A), xylitol (B), and HAMF (C), on sensory attributes, texture, and digestibility (dependent variables) of cookies. The central composite design was used, and the experimental design was generated by Design‐Expert (Stat‐Ease). After selecting the approximate range for each independent variable by preliminary experiments, the final experimental design was confirmed and is shown in Table 1. The data of three types of responses, including sensory attributes (appearance, color, flavor, taste, mouthfeel, and overall acceptability), texture (hardness and flexibility), and digestibility (soluble starch and resistant starch), are shown in Table 2 and were statistically analyzed by Design‐Expert. The independent and dependent variables were fitted by a second‐order model equation, and the goodness of fit was examined. Analysis of variances was conducted to analyze the lack of fit and the significance of the linear, quadratic, and interaction effects of the independent variables on each response. The optimized amounts of butter, xylitol, and HAMF for preparing cookies with optimal responses were calculated as described (Yun et al., 2008).

**Table 1:** Experimental design for low‐sugar cookies with different levels of butter, xylitol, and HAMF

Trial	A: Butter (g)	B: Xylitol (g)	C: HAMF (g)
1	225	150	250
2	150	100	250
3	150	100	250
4	75	50	250
5	225	100	350
6	225	100	150
7	150	50	350
8	150	100	250
9	150	150	150
10	150	100	250
11	75	100	150
12	150	100	250
13	75	150	250
14	150	150	350
15	75	100	350
16	150	50	150
17	225	50	250

**Table 2:** Response data of low‐sugar cookie under different conditions of butter, xylitol, and HAMF by central composite design

Parameters	Trial																
	1	2	3	4	5	6	7	8	9	10	11	12	13	14	15	16	17
Sensory evaluation																	
Appearance	6.143	6.557	6.543	4.983	6.686	6.257	5.629	6.446	6.391	6.257	4.871	6.420	5.331	6.029	5.346	4.869	5.671
Color	6.457	6.557	6.657	5.817	6.443	6.060	6.029	5.657	6.629	6.129	5.371	5.889	5.543	6.086	5.514	5.100	5.786
Flavor	6.086	5.700	6.114	4.806	5.757	5.686	5.257	6.346	6.400	5.909	4.971	5.914	5.460	6.071	5.363	5.257	5.631
Taste	5.870	5.672	5.820	4.342	5.804	5.339	4.507	5.868	6.683	5.825	4.431	5.796	5.502	5.992	4.918	4.704	4.433
Mouthfeel	6.571	5.863	5.751	4.154	6.186	6.206	5.057	5.477	6.403	5.580	3.900	5.483	5.057	5.914	4.623	5.726	5.889
Overall acceptability	5.757	5.927	6.045	3.806	5.575	5.641	5.035	6.322	6.450	5.956	3.798	5.941	4.901	5.807	4.348	4.856	4.781
Texture																	
Hardness (g)	1821.9	1911.4	1858.6	5339.7	1504.4	2249.6	2004.7	2398.0	2974.7	2139.0	6205.8	2033.8	4249.3	2173.4	3707.7	2153.0	1490.2
Flexibility (mm)	1.724	1.846	1.372	2.384	1.937	1.358	2.053	2.460	2.223	2.272	2.976	2.506	1.424	1.774	2.314	2.765	2.602
Digestibility																	
SS content (%)	43.244	49.738	45.620	55.330	39.728	52.421	46.043	46.551	52.920	39.512	61.353	48.246	51.464	38.083	49.472	53.237	47.220
RS content (%)	17.680	18.906	18.105	18.028	19.373	18.065	16.353	18.827	17.719	18.402	19.692	20.467	17.661	18.613	18.100	17.073	19.422

Note

Trials 1‐17 are cookies prepared by different levels of butter, xylitol, and HAMF according to the experimental design in Table 1.

Abbreviations: HAMF, high‐amylose maize flour; RS, resistant starch.SS, soluble starch.

## 3 RESULTS AND DISCUSSION

### 3.1 Chain length distribution of high‐amylose maize starch

**Figure 1:**
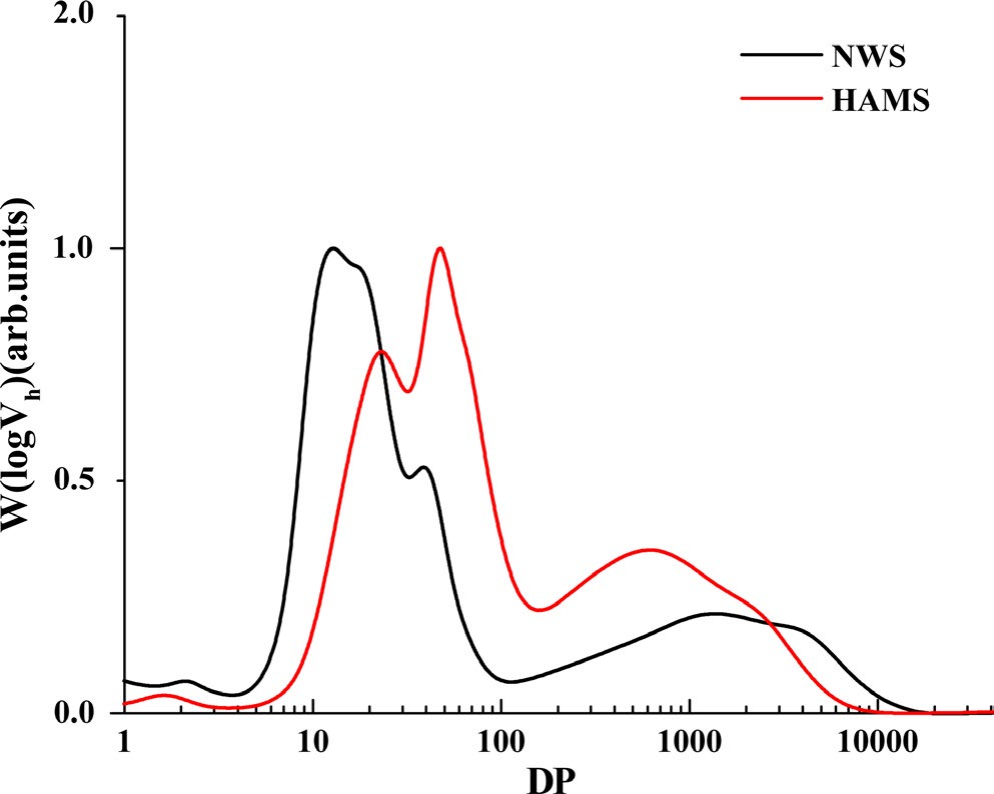
Weight distribution of debranched normal wheat starch (NWS) and high‐amylose maize starch (HAMS)

Typical GPC weight distributions, W(logV_h_), of individual chains obtained from debranched NWS and HAMS are shown in Figure 1, after normalizing to the peak maximum. The components of DP <100 are defined as amylopectin chains and those of DP >100 are amylose chains (Bertoft, 2017). NWS and HAMS both showed two peaks of AP branches and a peak of AM branches. The first AP peak (AP1) was composed of the short amylopectin branches with DPs ranging from 3 to 36, and the second peak (AP2) was composed of long amylopectin branches with DP lengths from 36 to 100 (Xu et al., 2017). HAMS showed higher peaks of AP2 and AM and lower peaks of AP1 than NWS, indicating higher proportions of long amylopectin and amylose chains and lower short amylopectin chains in HAMS (Xu et al., 2017). Moreover, AP1 and AP2 peaks of HAMS exhibited higher DP than NWS, suggesting a smaller molecular size of short and long amylopectin chains of HMAS; however, the lower DP of HAMS in AM peaks also suggested its smaller amylose molecular size.

### 3.2 Evaluation of fitted model

After acquiring all data of responses (sensory profiles, texture properties, and digestibility), a mathematical equation was fitted by Design‐Expert to describe the behavior of the responses. To evaluate the reliability of the model, analysis of variance (ANOVA) was conducted. Briefly, a reliable model should display a significant regression and a nonsignificant lack of fit (Bezerra, Santelli, Oliveira, Villar, & Escaleira, 2008). The former can be used to describe the major part of the variation, and the latter can be used to observe the remainder of the variation (Bezerra et al., 2008). The model equation and regression of all responses, including sensory attributes (appearance, color, flavor, taste, mouthfeel, and overall acceptability), texture (hardness and flexibility), and digestibility (soluble starch and resistant starch), were analyzed by Design‐Expert and are presented in Table 2. Further analysis of the significance of independent variables to responses and lack of fit is shown in Table 3. On the basis of the principle of significance in regression (*p* < .05) and nonsignificance in lack of fit (*p* > .05), it was concluded that the quadratic models for appearance, flavor, taste, mouthfeel, overall acceptability, hardness, soluble starch content, and resistant starch content were reliable. The models for color and flexibility were not reliable, and they were not further discussed below.

**Table 3:** Analysis of predicted model equation for the quality characteristics of low‐sugar cookies

Parameters	Model	*R* ^2^	*F*‐Value	*p*‐value Prob > *F*	Final equation in terms of coded factors:	
Appearance	Quadratic	0.9320	10.66	.0025	Appearance = +6.44 + 0.53 × A + 0.34 × B + 0.16 × C + 0.031 × A × B − 0.011 × A × C − 0.28 × B × C − 0.43 × A^2^ − 0.49 × B^2^ − 0.23 × C^2^	
Color	Quadratic	0.6293	2.83	.0706	Color = +5.98 + 0.31 × A + 0.25 × B + 0.11 × C + 0.24 × A × B + 0.060 × A × C − 0.37 × B × C	
Flavor	Quadratic	0.8745	5.42	.0183	Flavor = +6.00 + 0.32 × A + 0.38 × B + 0.017 × C − 0.050 × A × B − 0.080 × A × C − 0.082 × B × C − 0.40 × A^2^ − 0.099 × B^2^ − 0.15 × C^2^	
Taste	Quadratic	0.9011	7.09	.0086	Taste = +5.80 + 0.28 × A + 0.76 × B + 7.976E − 003 × C + 0.069 × A × B − 5.263E − 003 × A × C − 0.12 × B × C − 0.55 × A^2^ − 0.21 × B^2^ − 0.12 × C^2^	
Mouthfeel	Quadratic	0.8385	22.50	<.0001	Mouthfeel = +5.52 + 0.89 × A + 0.39 × B − 0.057 × C	
Overall acceptability	Quadratic	0.9610	19.16	.0004	Overall acceptability = +6.04 + 0.61 × A + 0.55 × B + 2.435E − 003 × C − 0.030 × A × B − 0.15 × A × C − 0.21 × B × C − 0.96 × A^2^ − 0.27 × B^2^ − 0.24 × C^2^	
Hardness	Quadratic	0.9590	18.17	.0005	Hardness = +2068.16 − 1554.55 × A + 28.96 × B − 524.11 × C + 355.52 × A × B + 438.23 × A × C − 163.25 × B × C + 1123.76 × A^2^ + 33.34 × B^2^ + 224.96 × C^2^	
Flexibility	Quadratic	0.3551	2.39	.1162	Flexibility = +2.12 − 0.18 × A − 0.33 × B − 0.16 × C	
SS content	Quadratic	0.7487	12.91	.0003	SS = +72.60 − 1.90 × A − 1.13 × B − 2.50 × C	
RS content	Quadratic	0.7487	12.91	.0003	RS = +27.40 + 1.90 × A + 1.13 × B + 2.50 × C	

Note

A = actual quantities of butter; B = actual quantities of xylitol; C = actual quantities of HAMF.

Abbreviations: HAMF, high‐amylose maize flour; RS, resistant starch; SS, soluble starch.

### 3.3 Effects of HAMF, butter, and xylitol on sensory properties of cookies

The models for appearance, flavor, taste, mouthfeel, and overall acceptability were all significant (*p* < .05), and their correlation coefficients were all higher than .80 (Table 3). Therefore, the proposed model is accurate for predicting changes in these responses within the experimental domain. As shown in Table 4, ANOVA demonstrated that the sensory attributes were significantly influenced by xylitol and butter (*p* < .05), while HAMF was not significant for these attributes (*p* > .05).

**Table 4:** ANOVA for response surface quadratic model

Source	*p*‐value									
	Appearance	Color	Flavor	Taste	Mouthfeel	Overall acceptability	Hardness (g)	Flexibility (mm)	SS content (%)	RS content (%)
Model	0.0025	0.0706	0.0183	0.0086	0.0023	0.0004	0.0005	0.2945	0.0016	0.0016
A	0.0005	0.0325	0.0078	0.0464	<0.0001	0.0002	<0.0001	0.2751	0.0029	0.0029
B	0.0058	0.0775	0.0031	0.0003	0.0069	0.0004	0.8504	0.0644	0.0415	0.0415
C	0.1060	0.3871	0.8520	0.9474	0.5995	0.9784	0.0094	0.3540	0.0004	0.0004
AB	0.8113	0.2140	0.6957	0.6865	0.7177	0.8170	0.1330	0.9293	0.6665	0.6665
AC	0.9291	0.7433	0.5351	0.9754	0.2442	0.2506	0.0744	0.2001	0.1172	0.1172
BC	0.0579	0.0659	0.5245	0.4789	0.7670	0.1387	0.4607	0.7768	0.1260	0.1260
Lack of fit	0.0326	0.7915	0.4707	0.0015	0.0665	0.1127	0.0399	0.6068	0.1851	0.1851

Abbreviations: HAMF, high‐amylose maize flour; RS, resistant starch; SS, soluble starch.

**Figure 2:**
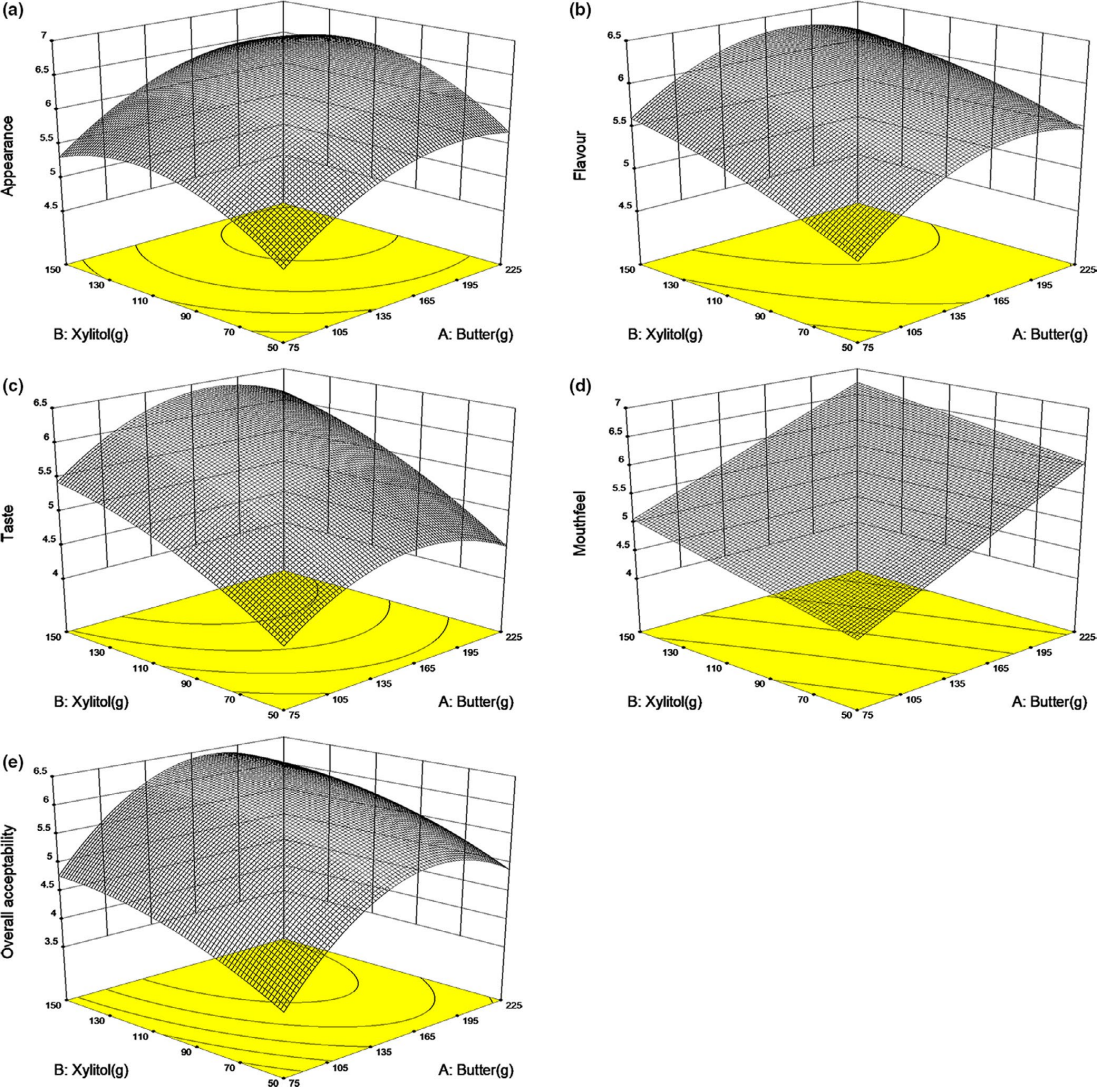
Response surfaces for the effect of butter and xylitol on sensory characteristics of low‐sugar cookies

The response surfaces of butter and xylitol on these sensory properties (Figure 2) show butter and xylitol were positively related to the appearance, mouthfeel, flavor, taste, and overall acceptability of cookies. Butter imparted shortening, richness, and tenderness during baking (Zoulias, Oreopoulou, & Tzia, 2002). Butter can prevent the interaction of the water or sugar solution with the flour protein, thereby influencing the continuity of the protein and starch structure and the textural properties of the cookie (Pareyt & Delcour, 2008). Xylitol is a good substitute for sucrose by providing the same texture and more finely taste than sucrose (Ur‐Rehman et al., 2015). The great potential of xylitol in producing bakery food with high acceptance has also reported (Kutyła‐Kupidura et al., 2016; Nourmohammadi & Peighambardoust, 2016).

### 3.4 Effects of butter, xylitol, and HAMF on the texture of cookies

**Figure 3:**
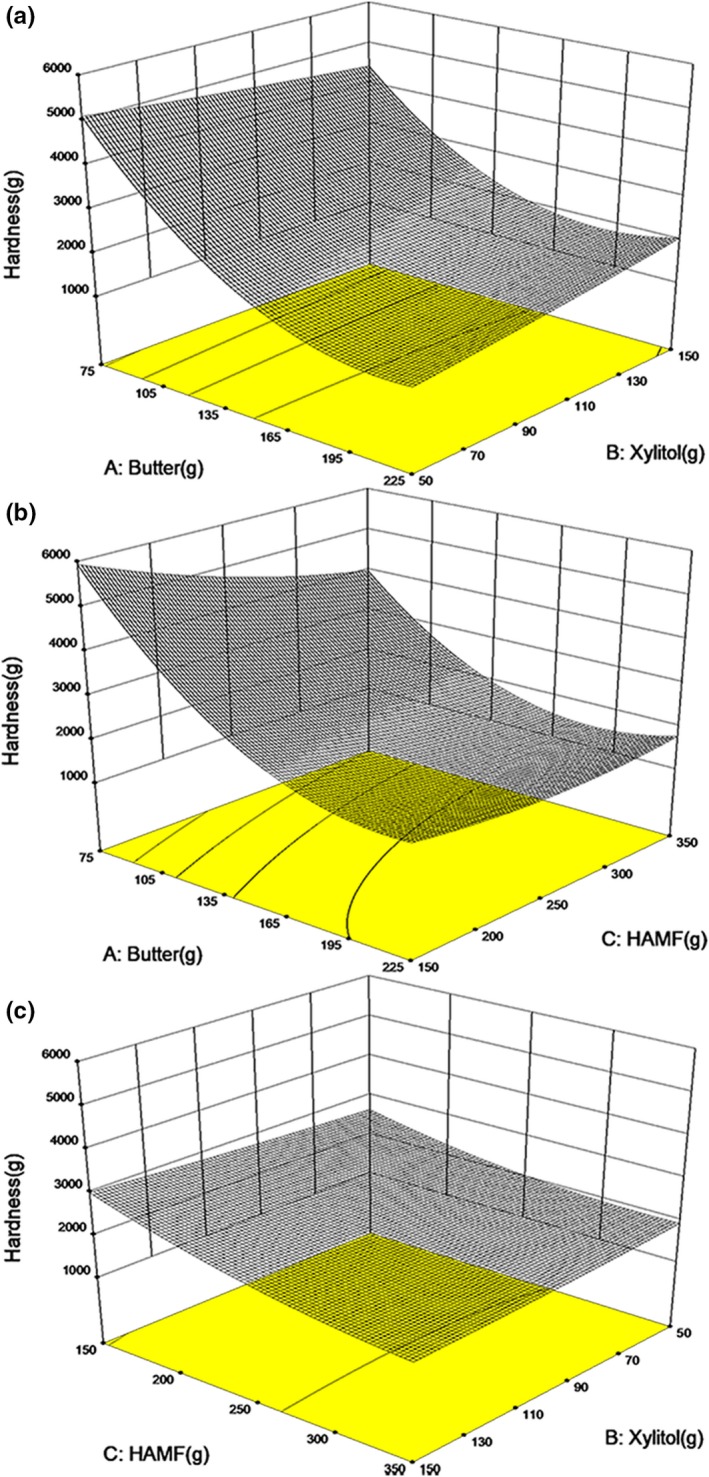
Response surfaces for the effect of butter and HAMF on texture of low‐sugar cookies

The model of hardness here was significant here, with a correlation coefficient of .9590 and a *p*‐value <.005 (Table 3). ANOVA suggested that hardness was mainly influenced by butter and HAMF (*p* < .05), while xylitol was not significant here (Table 4). Both butter and HAMF were negatively correlated with the hardness of cookie (*p* < .05) (Figure 3). As mentioned (Pareyt & Delcour, 2008), butter prevented the continuity of the protein and starch structure, thereby decreasing the hardness of cookies. It is important to mention that HAMF had the negative effect on hardness of cookies in this butter–xylitol–HAMF system. It is already known the significant effects of HAMS on increasing hardness of bakery products by recrystallization of amylose chains (Giuberti et al., 2016; Hung et al., 2005; Zhong et al., 2018). The decrease of hardness in HAMF may be attributed to the lower gluten content in the cookie. Gluten, the main component of wheat flour, is responsible for building structures of cookie and providing good gas‐holding ability and crumb structure of cookie (Gallagher et al., 2004). Fustier et al. (2008) studied the effects of gluten, starch, and water‐soluble fractions in wheat flour on quality of semi‐sweet biscuit, and found hardness of biscuit was mainly impacted by gluten concentration. HAMF is gluten‐free, and thus, using HAMF as substitution of wheat flour reduced the gluten concentration in dough inducing decreased hardness of cookie, although starch in HAMF may offset the decrease in the hardness to some content.

### 3.5 Effects of butter, xylitol, and HAMF on digestibility of cookies

**Figure 4:**
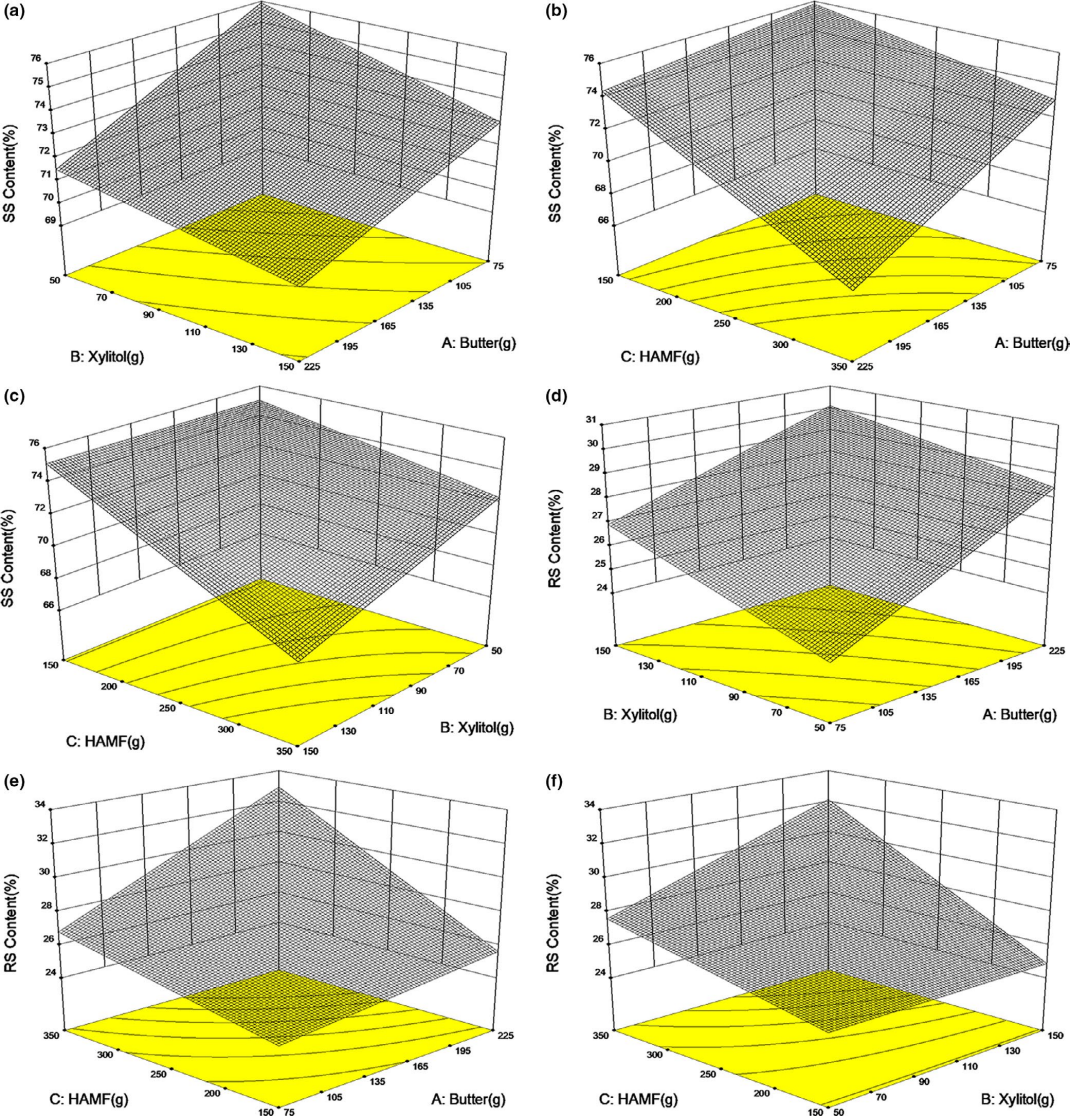
Response surfaces for the effect of butter, xylitol, and HAMF on digestibility of low‐sugar cookies

ANOVA showed models of soluble starch (SS) and resistant starch (RS) were both reliable (Table 2), and butter, xylitol, and HAMF were all significant for SS and RS (Table 3), within the experimental domain. Contents of SS and RS are negatively correlated, so that only RS was discussed to explain the changes in digestibility. Results showed butter, xylitol, and HAMF were all positively related to the RS content of cookies (Figure 4).

During the baking process, starch underwent gelatinization and retrogradation (Le‐Bail, Hesso, & Le‐Bail, 2018). During gelatinization, granular and crystalline structures of starch were disrupted. During retrogradation, starch molecules recrystallized and RS III formed. The formation of RSIII is highly related to the proportions of amylose and long amylopectin chains (You, Oh, Kim, & Chung, 2015). The molecular structure analysis (Figure 1) showed HAMS had higher amounts of long amylopectin and amylose chains than NWS, suggesting more enzymatic‐resistant double‐helix structure formed by amylose chains and long amylopectin chains during cooling process of baking (Jane, 1984; Witt, Gidley, & Gilbert, 2010). V‐type crystalline, which is complexes of amylose and lipid, was also formed during baking HAMF cake (Zhong et al., 2018).

The effect of xylitol on affecting RS content is possibly related to its ability on restricting the swelling of starch granules and retarding the gelatinization of starch (Martínez, Pico, & Gómez, 2015), by preventing the disruption of partial RS II residues (semi‐crystalline structure in HAMS) (Jiang, Campbell, Blanco, & Jane, 2010).

Butter is mainly composed of fatty acid and triacylglycerol (Foubert, Vanrolleghem, Thas, & Dewettinck, 2004). Amylose can form amylase‐resistant single‐helical complexes with fatty acid and triacylglycerol, especially in HAMS (Ai, Hasjim, & Jane, 2013). These complexes (RS V) can contribute to substantially less postprandial plasma glucose and insulin responses in human subjects (Hasjim et al., 2010).

### 3.6 Optimal combinations of butter, xylitol, and HAMF on producing low‐sugar cookie

The optimum parameters obtained from RSM can be used to achieve any given functional properties. As shown in Table 5, the optimal amounts of butter, xylitol, and HAMF to obtain cookies with the highest sensory acceptability, the highest RS content, the lowest hardness, and their combinations were selected through numerical optimization of a canonical model by Design‐Expert. The optimal amounts of three ingredients to prepare a cookie with minimum hardness and maximum overall sensory acceptability and RS content are 183 g butter, 150 g xylitol, and 341 g HAMF, respectively.

**Table 5:** Optimal parameters of low‐sugar cookies with highest overall acceptability, RS content, or the combinations of them

	Butter (g)	Xylitol (g)	HAMF (g)
Overall acceptability (maximize)	176	150	196
Hardness (minimize)	214	50	247
RS content (maximize)	225	150	350
Overall acceptability (maximize) and hardness (minimize)	180	138	258
Overall acceptability (maximize) and RS content (maximize)	188	150	334
Hardness (minimize) and RS content (maximize)	207	150	350
Overall acceptability (maximize), hardness (minimize), and RS content (maximize)	183	150	341

Abbreviations: HAMF, high‐amylose maize flour; RS, resistant starch; SS, soluble starch.

### 3.7 Functions of HAMF on a cookie‐baking system containing butter and xylitol

Effects of HAMS and/or HAMF on bakery foods have been widely studied in bread (Hoebler, Karinthi, Chiron, Champ, & Barry, 1999), cake (Zhong et al., 2018), and cookie (Luhovyy et al., 2014). Adding HAMS and HAMF can significantly increase RS content of these foods (Hoebler et al., 1999; Luhovyy et al., 2014; Zhong et al., 2018). However, the replacement of wheat flour with HAMS and HAMF decreased the sensory attributes and texture of these bakery products to some content in some studies (Giuberti et al., 2016; Luhovyy et al., 2014), thereby affecting the development of high‐amylose maize in the food industry. The results suggested HAMF did not affect sensory profiles of cookie significantly when butter and xylitol were also set as variables. The information implies HAMF/HAMS had insignificant effects on decreasing sensory properties and increasing hardness of cookie through introducing other important ingredients for sensory properties and hardness, such as butter and xylitol.

It is worth noting that HAMF content was negatively related to the hardness of cookie. In our hypothesis, the hardness of cookie should decrease after adding HAMF due to increased recrystallization of amylose chains from HAMS. The converse result suggests that HAMS was not the main factor affecting the texture of cookie, while gluten‐free characteristic of HAMF may be responsible for the decrease in hardness. The increase in the cookie texture after adding HAMF was also found in another study (Giuberti et al., 2016).

High‐amylose maize flour, butter, and xylitol all increased RS content of cookies. Due to the higher amylose and long amylopectin contents of HAMS than NWS, high proportions of enzymatic‐resistant double helices formed during baking of HAMF‐based cookie. Moreover, during baking, xylitol restricted the swelling of starch granules and retarded the gelatinization, and protected some RS II residues. Butter increased RS by forming enzymatic‐resistant complexes with amylose. The results imply the interactions among HAMF, xylitol, and butter in the cookie, resulting in the increasing of RS content.

Overall, in the cookie system containing butter and xylitol, HAMF was a potential ingredient to partially replace normal wheat flour. Analysis of optimal combinations of butter, xylitol, and HAMF (Table 5) showed there were various combinations of three ingredients to produce cookies with different attributes. For instance, when the purpose was only to maximize the sensory attributes of the cookie, the combination of ingredients was predicted as intermediate butter content (176 g), high xylitol content (150 g), and low HAMF content (196 g); while the purpose was to maximize sensory acceptability, minimize hardness, and maximize RS content meanwhile, the combination was intermediate butter content (183 g), high content of xylitol (150 g), and high HAMF content (341 g). In this system, with the addition of HAMF, RS content increased, and hardness decreased. The increase in RS content was due to the high‐amylose content of HAMF, and the decrease in hardness was related to the gluten‐free characteristics of HAMF. Sensory attributes of the cookie can be significantly improved by adding butter and xylitol, while adding HAMF had no effects on sensory properties. However, in bakery systems without ingredients such as butter and xylitol, HAMF/HAMS had significant effects on decreasing sensory attributes and increasing hardness of bakery foods (Giuberti et al., 2016; Hung et al., 2005; Zhong et al., 2018). Therefore, we suggest butter and xylitol can be added as other ingredients when HAMF‐based cookies are prepared, in order to improve sensory properties and decrease hardness.

## 4 CONCLUSION

We studied the effects of butter, xylitol, and high‐amylose maize flour (HAMF) on sensory attributes, texture, and digestibility of cookie by response surface experimental design. Results showed that butter and xylitol were significant for sensory profiles; butter and HAMF were mainly responsible for hardness; and butter, xylitol, and HAMF were all significant for RS content in the cookie. The digestibility of cookie was mainly related to the molecular structure of HAMS, which has high amounts of amylose and long amylopectin chains. The optimal conditions of butter, xylitol, and HAMF content were also predicted to obtain cookie with highest sensory attributes and RS content and lowest hardness. These results suggest that HAMF had an insignificant influence on sensory properties and hardness of cookie and significant effect on RS content in complex baking process within butter and xylitol, which is important for the development of low‐sugar foods and the application of high‐amylose maize on the food industry.

## CONFLICT OF INTEREST

The authors declare that they do not have any conflict of interest.

## ETHICAL APPROVAL

This study does not involve any human or animal testing.
